# Regulation of Nuclear NF-κB Oscillation by a Diffusion Coefficient and Its Biological Implications

**DOI:** 10.1371/journal.pone.0109895

**Published:** 2014-10-10

**Authors:** Daisuke Ohshima, Kazuhisa Ichikawa

**Affiliations:** Division of Mathematical Oncology, The Institute of Medical Science, The University of Tokyo, Minato-ku, Tokyo, Japan; Academia Sinica, Taiwan

## Abstract

The transcription factor NF-κB shuttles between the cytoplasm and the nucleus, and nuclear NF-κB is known to oscillate with a cycle of 1.5-2.5 h following the application of external stimuli. Oscillation pattern of NF-κB is implicated in regulation of the gene expression profile. In a previous report, we found that the oscillation pattern of nuclear NF-κB in a computational 3D spherical cell was regulated by spatial parameters such as nuclear to cytoplasmic volume ratio, nuclear transport, locus of protein synthesis, and diffusion coefficient. Here we report analyses and a biological implication for the regulation of oscillation pattern by diffusion coefficient. Our analyses show that the “reset” of nuclear NF-κB, defined as the return of nuclear NF-κB to the initial level or lower, was crucial for the oscillation; this was confirmed by the flux analysis. In addition, we found that the distant cytoplasmic location from the nucleus acted as a “reservoir” for storing newly synthesized IκBα. When the diffusion coefficient of proteins was large (≥10^−11^ m^2^/s), a larger amount of IκBα was stored in the “reservoir” with a large flux by diffusion. Subsequently, stored IκBα diffused back to the nucleus, where nuclear NF-κB was “reset” to the initial state. This initiated the next oscillation cycle. When the diffusion coefficient was small (≤10^−13^ m^2^/s), oscillation of nuclear NF-κB was not observed because a smaller amount of IκBα was stored in the “reservoir” and there was incomplete “reset” of nuclear NF-κB. If the diffusion coefficient for IκBα was increased to 10^−11^ m^2^/s keeping other proteins at 10^−13^ m^2^/s, the oscillation was rescued confirming the “reset” and “reservoir” hypothesis. Finally, we showed altered effective value of diffusion coefficient by diffusion obstacles. Thus, organelle crowding seen in stressed cells possibly changes the oscillation pattern by controlling the effective diffusion coefficient.

## Introduction

NF-κB is a transcription factor regulating more than 500 genes [1]. It is activated by extracellular stimuli including proinflammatory cytokines, viral infection and cell stress [2]–[11]. On activation, NF-κB translocates from the cytoplasm to the nucleus, and back again. If the activating stimulus continues, activated NF-κB shuttles back and forth between the cytoplasm and the nucleus, and thus an oscillation of nuclear NF-κB (NF-κB_n_) is observed [12]–[14]. Importantly, different oscillation patterns are implicated in different gene expression profiles [13]. It is also reported that NF-κB in cancer cells is constitutively active, and that its hyperesponsiveness leads to autoimmune diseases [15], [16]. Thus, a proper regulation of NF-κB is crucial for the cell's fate.

The mechanisms regulating NF-κB have been extensively studied. In the canonical NF-κB pathway, TNF/IL-1 receptors, IRAK1/4, TRAF2/5/6, Ubc13/Uev1A, Tak1, TAB1/2/3, MEKK3, IKKα/IKKβ/NEMO (IKKγ), IκBα/β/ε, Bcl3, A20, CYLD, PKA, and PP2A are found to be involved in the regulation of the NF-κB family member p50:p65 (p50:RelA) complex [3], [4], [17], [18]. In addition, phosphorylation, ubiquitination, proteasomal degradation, and de novo protein synthesis play crucial roles in the activation and regulation of NF-κB [4], [19], [20]. If some of these molecules and/or posttranscriptional modifications are abrogated, NF-κB activity is dysregulated. In fact, knockout of IκBα, IκBβ, or IκBε led to an altered oscillation pattern of NF-κB_n_ [12]. Innate immune responses were reported to be deficient in p50 knockout mice [21]. Nuclear translocation of NF-κB was severely impaired in TRAF2 and TRAF5 double knockout mice [22]. In TAK1-deficient mouse embryonic fibroblasts (MEFs), TRAF6 did not bind MEKK3 [17], and MEKK3 knockout mice were unable to degrade IκBα following TNFα stimulation [23]. IKKβ-deficient cells had impaired cytokine-induced NF-κB activation [24], [25]. A20-deficient cells cannot properly terminate TNF-induced NF-κB activity [26]. Bcl3 is a nuclear member of the IκB family, and its deficiency leads to a hypersensitivity to cytokine stimulation [27]. All these studies clearly show the important role of these molecules and posttranslational modification in the regulation of NF-κB.

The cell is a three-dimensional (3D) entity with complex and complicated internal structures known as organelles. In a cancer cell, it is known that the size of the nucleus increases as the malignancy progresses [28]–[30]. It is also well known that the shape of the nucleus is aberrant in progeria patients [31], [32]. In addition, the density of nuclear pores on the nuclear envelope was reported to be increased in malignant cancer cells [33]–[35]. It was also reported that the density differed according to the type of melanoma cell [36]. Furthermore, mitochondria were reported to crowd around the nucleus upon hypoxia in pulmonary artery endothelial cells [37]. The distribution of mitochondria is also changed by viral infection [38]. Both hypoxia and viral infection activate NF-κB [5]–[10]. Thus, the structure of intracellular space, that is the population, density, or localization of organelles, is changed by NF-κB-activating stimuli. Although these observations suggest a role of intracellular structure on the regulation of NF-κB activity, it is not known whether the change in the intracellular structure has any effect on the oscillation pattern of NF-κB.

In a previous report, we developed a 3D computational model of NF-κB activation showing the effects of spatial parameters including nuclear to cytoplasmic volume ratio (N/C ratio), transport through nuclear envelope, locus of protein synthesis, and diffusion coefficient, on the oscillation pattern of NF-κB [39]. Here we report a detailed analysis of the mechanism of the alteration in the oscillation pattern by a diffusion coefficient. Firstly, we show a bifurcation in the NF-κB oscillation following a change in the diffusion coefficient. Further analysis shows that the “reset” of NF-κB_n_ is a key mechanism for the oscillation. A large diffusion coefficient contributes to the “reset” of NF-κB_n_ by storing IκBα in a distant location in the cytoplasm and by the subsequent replenishment of the nucleus by a large influx of IκBα. Thus, the cytoplasm acts as “reservoir” for IκBα. Finally we show simulations suggesting that the change in the distribution of organelles alters the effective value of the diffusion coefficient, and thereby changes the oscillation pattern of NF-κB_n_.

## Results

### Altered oscillation pattern of NF-κB_n_ due to a change in the diffusion coefficient

**Figure 1:**
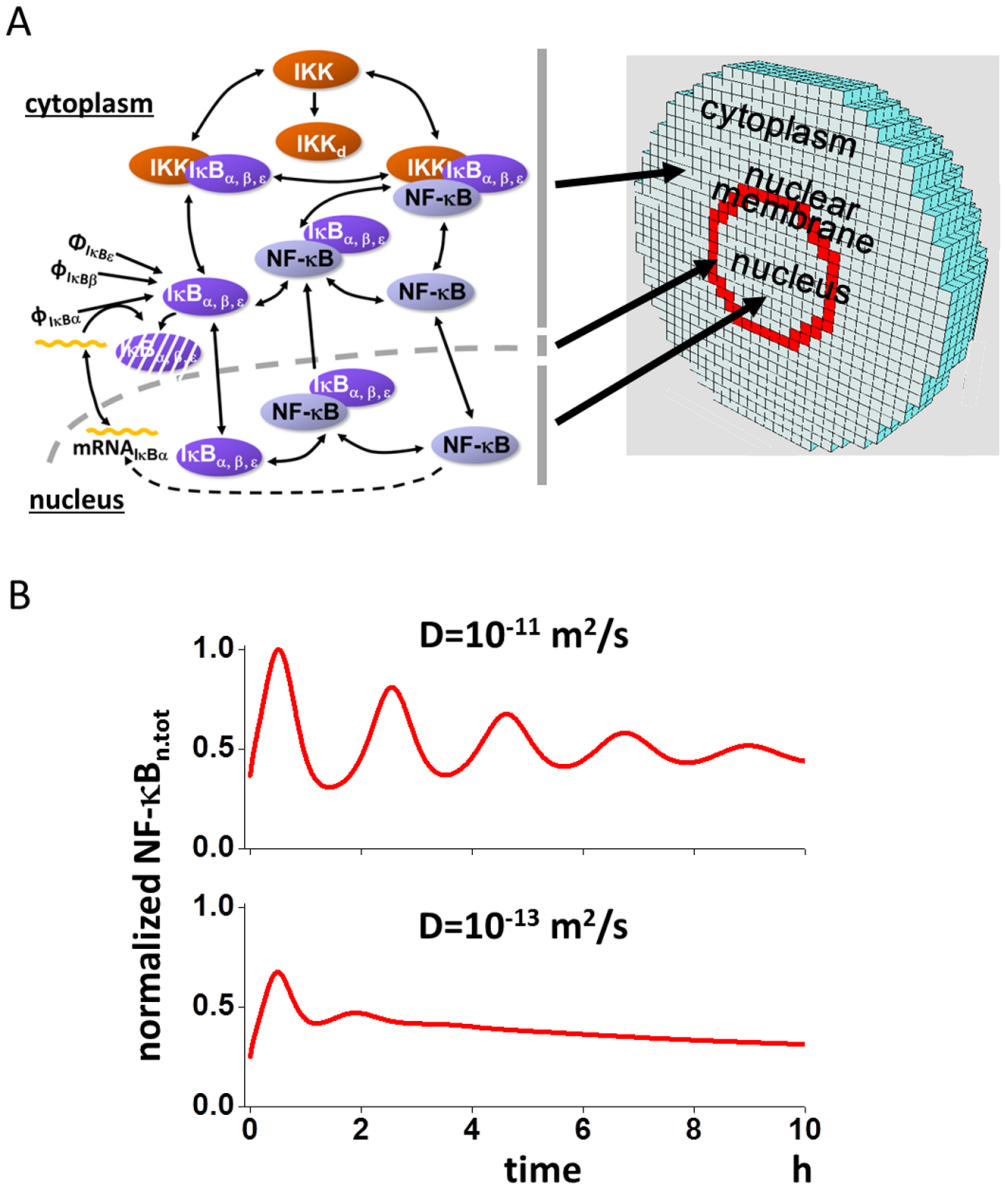
Oscillation pattern of nuclear NF-κB is altered by the change in diffusion coefficient. (A) Reaction schemes and the shape of the 3D model were the same as in a previous report [39], and the detailed reaction scheme and parameter values are shown in Figure S1 and Table S1. The 3D model had a spherical shape with a diameter of 50 µm, which was divided into 62,417 cubic compartments allowing reaction-diffusion simulations. Reaction schemes were embedded into the corresponding region of the cytoplasm, nuclear membrane (red compartments) and nucleus. (B) The effect of the diffusion coefficient on the oscillation pattern of NF-κB_n.tot_. NF-κB_n.tot_ was the summation of the concentrations of free NF-κB_n_ and IκB_n_:NF-κB_n_ in the nucleus, which corresponded to the fluorescent light intensity in the experiments. While NF-κB_n.tot_ oscillated at D for proteins of 10^−11^ m^2^/s, it did not oscillate at a smaller D for proteins of 10^−13^ m^2^/s.

We used a 3D model to investigate alterations in the oscillation pattern of NF-κB_n_ (Figure 1A) [39]. In short, activated IKK binds to the complex of IκB (IκBα, IκBβ, or IκBε) and NF-κB (IκB:NF-κB) leading to the phosphorylation of IκB and subsequent proteasomal degradation. NF-κB, ”liberated” as a result of IκB degradation, translocates to the nucleus, where it promotes the expression of the IκBα gene. The IκBα mRNA thus generated is exported from the nucleus to the cytoplasm, where IκBα is newly synthesized and then translocates back to the nucleus. This facilitates the formation of the IκBα:NF-κB complex in the nucleus, and NF-κB is exported back to the cytoplasm. These reaction schemes were embedded to the corresponding regions, that are the cytoplasm, nuclear membrane, and nucleus, of a spherical 3D model cell of 50 µm diameter and an N/C ratio of 8.3% [39], [40]. The 3D model cell was divided into 62,417 small compartments of identical size allowing reaction-diffusion simulations. Diffusion between adjacent compartments was calculated by Fick's equation. Red compartments in Figure 1A indicate the nuclear membrane. A detailed description of the reaction schemes is shown in Figure S1A, and all parameters for simulations are listed in Table S1.

We employed diffusion coefficient (D) of 10^−11^ and 10^−13^ m^2^/s for proteins (D_protein_) and mRNA (D_mRNA_), respectively [39], [41]–[45]. At these values of D_protein_ and D_mRNA_, simulated NF-κB oscillation replicated the same observation previously reported in experiments with fluorescence-labeled NF-κB [13] (upper panel of Figure 1B). We employed total NF-κB_n_ (NF-κB_n.tot_) to show the oscillation of nuclear NF-κB, which is the summation of free NF-κB_n_ and the nuclear complex of IκB_n_:NF-κB_n_, because in the experiments using fluorescence-labeled NF-κB, total fluorescence was measured. When D_protein_ was reduced to 10^−13^ m^2^/s keeping D_mRNA_ unchanged, virtually no oscillation of NF-κB_n.tot_ was seen (lower panel of Figure 1B). Thus, the change in D_protein_ alters the oscillation pattern of NF-κB_n.tot_ as previously reported [39].

### Oscillation of NF-κB_n.tot_ shows bifurcation-like behavior in response to a change in D_protein_

**Figure 2:**
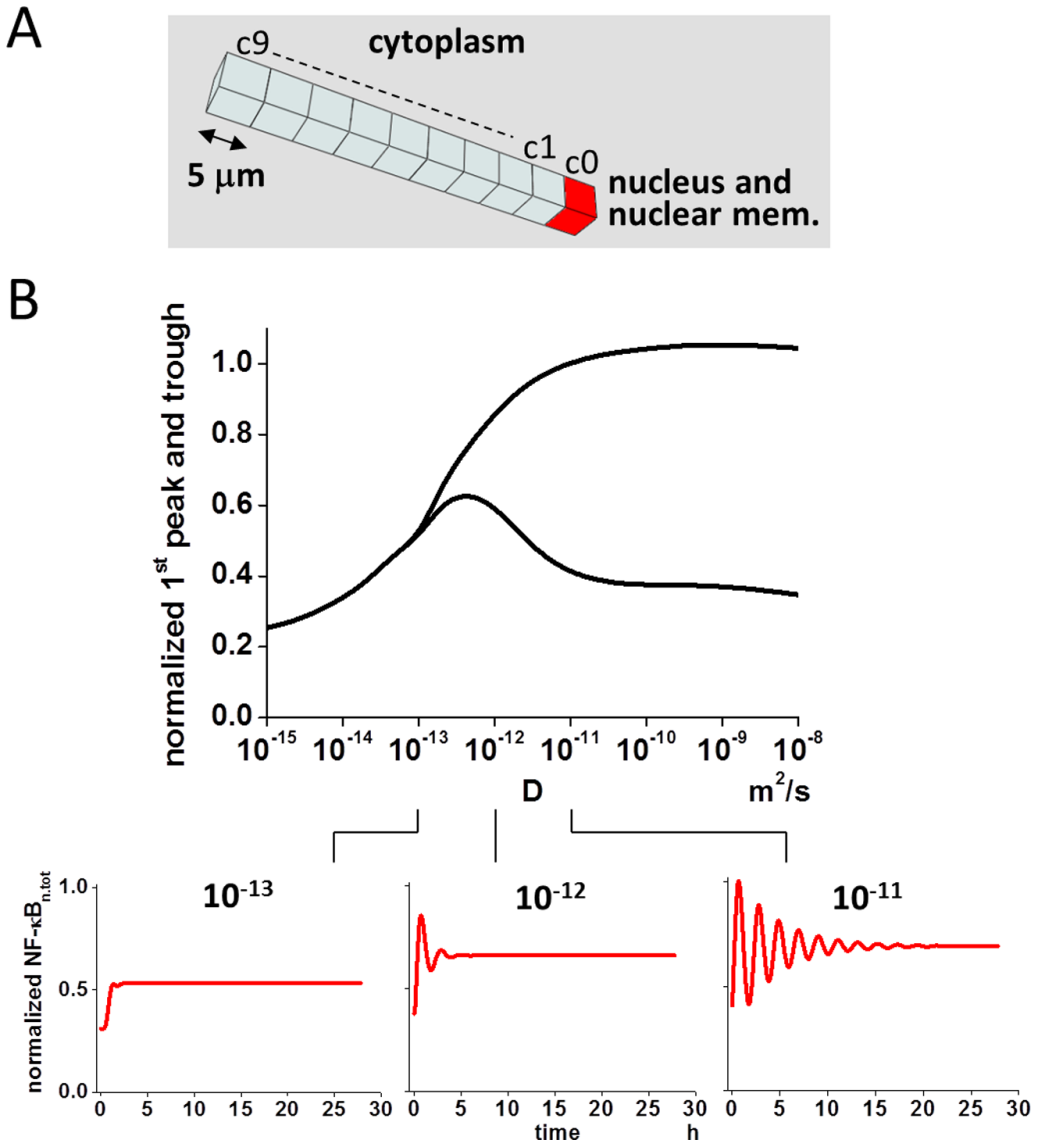
Bifurcation-like regulation of nuclear NF-κB oscillation by diffusion coefficient. (A) To investigate the effect of a diffusion coefficient on the oscillation of NF-κB_n.tot_, a simple 1D model was constructed. There were 10 cubic compartments (c0, c1,---,c9), measuring 5 µm along each side, and the rightmost compartment was assigned as the nucleus and nuclear membrane (red cube). (B) Oscillation was investigated with a wide range of diffusion coefficient of proteins from 10^−15^ to 10^−8^ m^2^/s. By plotting the concentration of NF-κB_n.tot_ at the first peak and trough, a bifurcation-like diagram was clearly seen. At D lower than 10^−12^ m^2^/s, oscillation was not observed, while at D higher than this, oscillation was observed, becoming more pronounced at higher D.

To see the change in the oscillation of NF-κB_n.tot_ over wider range of D_protein_, and to analyze its mechanisms, we constructed a simple 1D model (Figure 2A and Figure S1B). In this 1D model, there were 10 cubic compartments of identical size (length of edge: 5 µm); one of the 10 was assigned as the nucleus and nuclear membrane (red cubic compartment in Figure 2A).

We ran simulations by changing D_protein_ from 10^−15^ to 10^−8^ m^2^/sec. Simulations under such a wide range of D_protein_ are helpful for elucidating the mechanisms for the regulation of NF-κB_n.tot_ oscillation by D_protein_. We defined NF-κB_n.tot_ as oscillating when there was at least one peak and trough in the time course of NF-κB_n.tot_ (Cf. Figure S2A). According to this definition, the concentrations of NF-κB_n.tot_ at the first peak and trough are shown in the upper panel of Figure 2B. It can be clearly seen that NF-κB_n.tot_ oscillated when D_protein_ was higher than 10^−12^ m^2^/s. At a lower D_protein_, NF-κB_n.tot_ did not oscillate. Thus, the oscillation of NF-κB_n.tot_ shows bifurcation-like characteristics. D_protein_ of 10^−12^ m^2^/s was a critical value because there was only one pronounced peak in the oscillation. Traditionally, bifurcation refers to system behavior near equilibrium. Although our analysis shown in Figure 1B was not based on equilibrium, the diagram resembles the same behavior as that drawn by the first peak and trough 20,000 sec after the activation of NF-κB (Figure S2B). The bifurcation was also observed in the original 3D model (Figure S3).

### Reset of nuclear NF-κB is crucial for the continued oscillation

**Figure 3:**
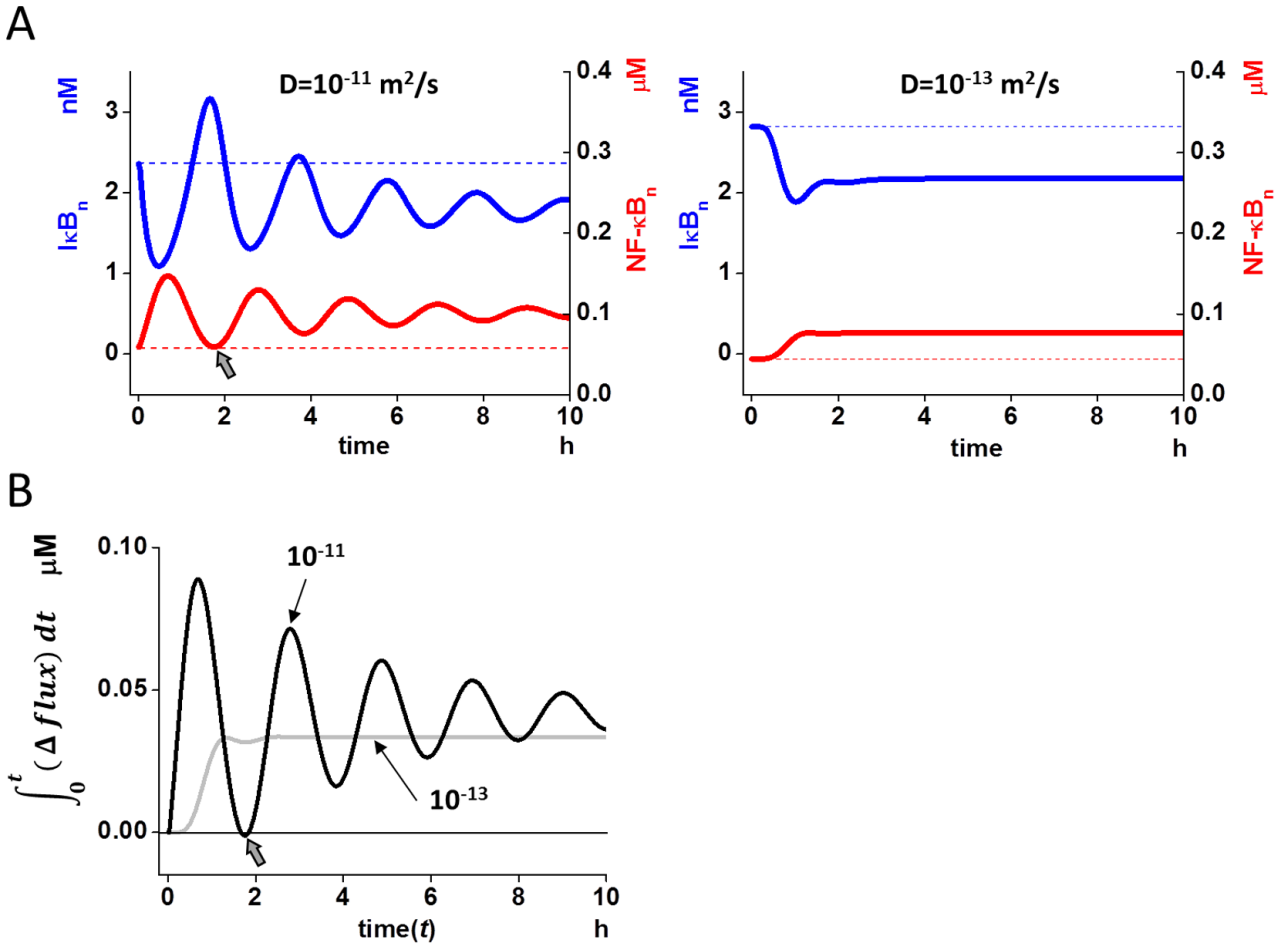
“Reset” of nuclear NF-κB by newly synthesized IκB is essential for the oscillation of nuclear NF-κB. (A) There was a difference in the nuclear IκB (IκB_n_) in oscillating (left) and non-oscillating (right) conditions of diffusion coefficient (10^−11^ and 10^−13^ m^2^/s, respectively). In the oscillating condition, peak IκB_n_ was higher than at the resting level (continuous and broken blue lines in the left panel), and NF-κB_n_ returned to the initial level, indicating the occurrence of a “reset” (gray arrow). In contrast, it was lower than the resting level in the non-oscillating condition (right panel). Red lines show free NF-κB_n_ for reference. (B) In oscillating D (10^−11^ m^2^/s, black line), cumulative *Δflux* (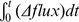), which is the integral of the difference in the inward fluxes of NF-κB and IκB to the nucleus from the start of the oscillation to time *t*, indicates a “reset” of the free NF-κB_n_ level to the initial state, because the cumulative *Δflux* reaches zero (gray arrow). Zero cumulative *Δflux* indicates the balance of inward fluxes between NF-κB and IκB, and all NF-κB that flowed into the nucleus is transported out of the nucleus at the time of zero cumulative *Δflux*. In contrast, the cumulative *Δflux* does not reach zero at non-oscillating D (10^-13^ m^2^/s, gray line), indicating the accumulation of NF-κB in the nucleus, and NF-κB_n_ is not reset.

Next we searched for a mechanism regulating NF-κB_n.tot_ oscillation by D_protein_. To this end, we compared the time courses of IκB_n_, because it is essential for the export of NF-κB_n_ from the nucleus, and incomplete export results in an accumulation of species leading towards system equilibrium. When NF-κB_n.tot_ was oscillating at a D_protein_ of 10^−11^ m^2^/s (left panel of Figure 3A), the first peak of IκB_n_ was higher than the initial level (blue continuous and broken lines) strongly suggesting the export of sufficient amount of free NF-κB_n_. In fact, free NF-κB_n_ reached its initial level at this time (gray arrow). Thus the system was “reset”, which we defined as the return of free NF-κB_n_ to the initial level or lower. In contrast, at D_protein_ of 10^−13^ m^2^/s, the first peak of IκB_n_ was lower than the initial level suggesting an insufficient export of NF-κB_n_ (blue continuous and broken lines in the right panel of Figure 3A). In fact, a considerable amount of free NF-κB_n_ remained in the nucleus at this time (red continuous and broken lines in the right panel of Figure 3A). Thus the system was not “reset” at a D_protein_ of 10^−13^ m^2^/s, and reached equilibrium quickly. The first peak of IκB_n_ is clearly shown in the magnified view (arrow in Figure S4A).

To further confirm this mechanism, we plotted cumulative *Δflux*, which was calculated by the following equations:



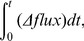
(1)


where the difference flux *Δflux* was calculated by



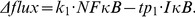
(2)


*k1•NFκB* and *tp1•IκB* are inward fluxes of NF-κB and IκB to the nucleus, respectively. According to the reaction schemes in the present model (Figure 1A and Figure S1B), if the cumulative *Δflux* is positive, the cumulative inward flux of NF-κB at *t* after its activation is larger than that of IκB indicating the higher free NF-κB_n_ concentration at *t* than the initial level. If it is 0, both fluxes are balanced indicating the same free NF-κB_n_ concentration at *t* as the initial level. As shown in Figure 3B, the cumulative *Δflux* for D_protein_ of 10^−11^ m^2^/s reached 0 (gray arrow) indicating a balance between NF-κB and IκB fluxes and “reset” to the initial level at the time point of the first trough of free NF-κB_n_. In contrast, it was positive at all time points for D_protein_ of 10^−13^ m^2^/s, indicating the excess inward flux of NF-κB, and no occurrence of “reset”. Thus, these analyses confirmed the “reset” mechanism of the system for the oscillation. Negative cumulative *Δflux* indicates lower free NF-κB_n_ concentration. In fact this was observed at D_protein_ of 10^−9^ m^2^/s at the first trough of free NF-κB_n_ (gray arrows in Figure S4B).

### Distant location in the cytoplasm acts as a reservoir for IκB

The next question was why the large D_protein_ caused the “reset” of NF-κB_n_ but the small D_protein_ did not. First we hypothesized that the difference in the homogeneity of the protein distribution by the difference in D_protein_ could have led to this difference. To test this possibility, we used the following equation:



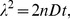
(3)


where *λ* ^2^ and *n* are mean square displacement and dimension (3 for 3D simulation). Using Eq.3, we could calculate *t*, which was a measure of the time required for the homogenous distribution within a space characterized by *λ*. The distance between the nuclear membrane and the plasma membrane in our spherical model cell (15 µm) gave *t* of 3.75 and 375 s for a D_protein_ of 10^−11^ and 10^−13^ m^2^/s, respectively. These values were considerably smaller than the oscillation period of NF-κB_n.tot_ (∼7,200 s). This indicates that proteins were distributed almost homogeneously during the period of oscillation in both cases. This strongly suggested that the difference in the inhomogeneity of protein distribution was not the reason for the difference observed between D_protein_.

The next question was what was the mechanism that led to the differences in oscillating and non-oscillating NF-κB_n.tot_ due to the difference in D_protein_? It should be noted that the flux by diffusion was calculated by the following equation:
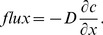
(4)


**Figure 4:**
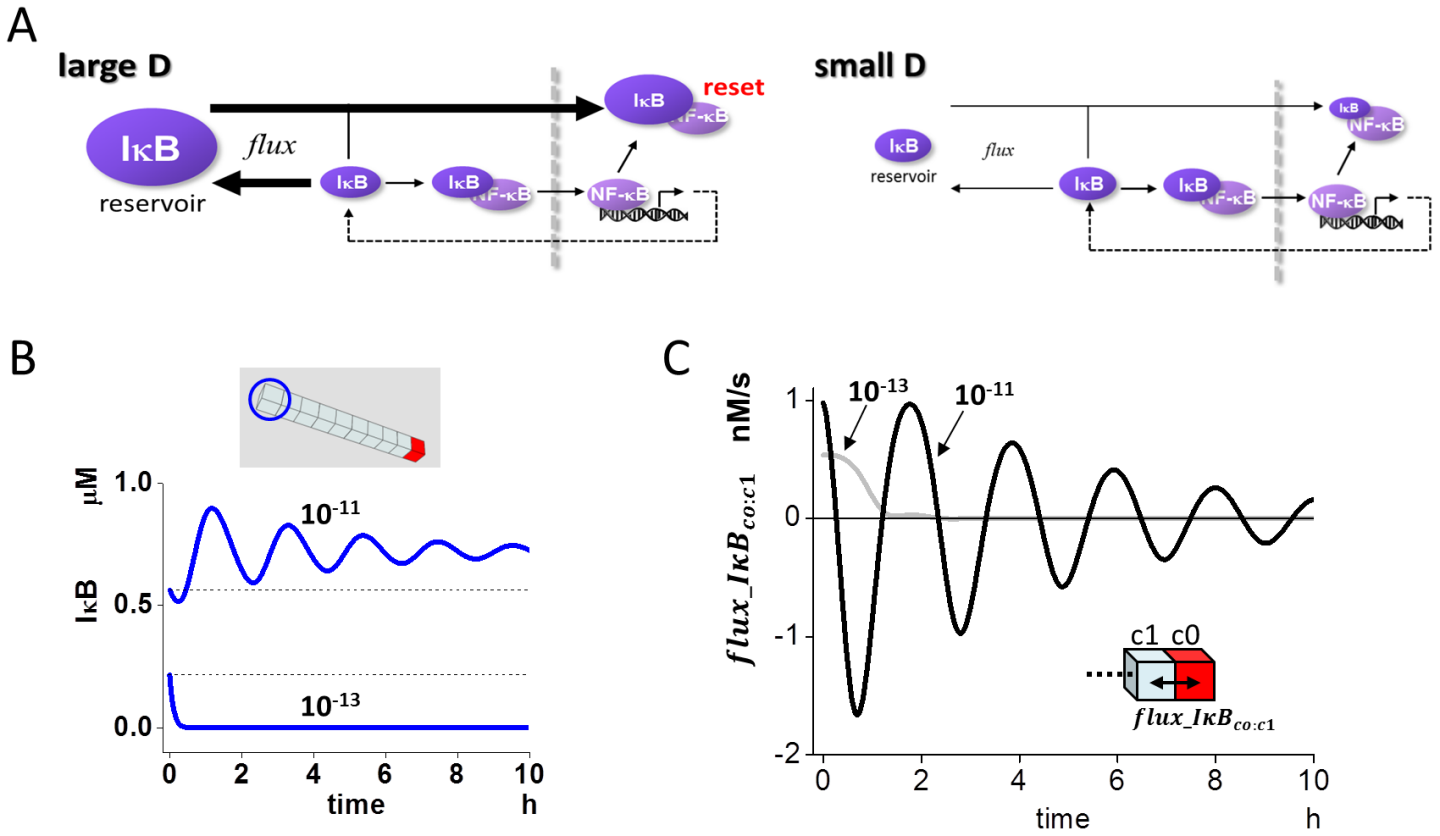
Storing IκB at a distant location in cytoplasm is critical for the oscillation. (A) A hypothesis why difference in the diffusion coefficient results in the difference in the “reset” state of NF-κB_n_. At large D, newly synthesized IκB was transported to a distant location in the cytoplasm with the large flux due to a large D, and the distant location acts as a “reservoir” for IκB. IκB in the “reservoir” diffused back to the nucleus in the subsequent time period, and was used to “reset” NF-κB_n_. In contrast at small D, the flux transporting IκB was small, and only a small amount of IκB was stored in the “reservoir”. This resulted in the imperfect “reset” of NF-κB_n_ reaching towards the equilibrium. (B) When D was large (10^−11^ m^2^/s), a considerable amount of IκB was stored at the distant compartment indicated by a blue circle (top panel). The peak concentration was significantly higher than the resting level (upper broken horizontal line in the bottom panel). In contrast, it was much lower at the distant compartment, when D was small (10^−13^ m^2^/s). These simulation results strongly support the hypothesis shown in (A). (C) Diffusion fluxes of IκB between c0 and c1 are shown. Fluxes relative to 10^5^ s after the start of the oscillation were measured to show net inward (negative value) and outward (positive value) flux to and from c0. At D of 10^−11^ m^2^/s, large inward fluxes were seen periodically indicating net inward flow from c1 to c0 (black line). On the contrary, only outward flux from c0 was seen at D of 10^−13^ m^2^/s indicating no replenishment to c0 from c1 in this small D condition (gray line).

While *λ* is proportional to the square root of D, flux by diffusion is proportional to D (Cf. Eqs.3 and 4). This indicates that the flux is more strongly affected by the change in D than *λ*. If D is 10-fold larger, the flux is also 10-fold larger indicating a 10-fold larger amount of proteins is transported to the distant location by diffusion. In light of this, we hypothesized that cytoplasmic location distant from the nucleus would act as a “reservoir” for IκB, where newly synthesized IκB is transported and stored. If D is large, a large amount of IκB will be stored in the “reservoir” and diffuse back to the nucleus with large flux, which can “reset” the activity of NF-κB_n_ (left panel of Figure 4A). In case of small D, a small amount of IκB will be stored in the “reservoir” and the flux back to the nucleus will also be small, which in turn will be insufficient to “reset” NF-κB_n_ (right panel of Figure 4A).

In fact, if we measured the concentration of IκB at the most distant compartment in a 1D model (the compartment c9 surrounded by a blue circle in Figure 4B), it was larger than the initial level at a D_protein_ of 10^−11^ m^2^/s. In contrast, it was much smaller at a D_protein_ of 10^−13^ m^2^/s. To further investigate the “reservoir” hypothesis, the diffusion flux of IκB between c0 and c1, *flux_IκB_co:c1_*, was measured (Figure 4C). However, the measurement of *flux_IκB_co:c1_* was not simply straightforward, because diffusion flux of IκB from c0 to c1 remained, even at equilibrium. This was because that there was a continuous degradation of IκB at c1, and a continuous supply of IκB from c0 to c1 was required to keep a balance with this degradation of IκB at c1 (Cf. Figures 1 and S1). Therefore, we calculated *flux_IκB_co:c1_* relative to the flux at equilibrium. Thus, *flux_IκB_co:c1_* was zero at equilibrium, and negative and positive values of *flux_IκB_co:c1_* indicated net inward and outward fluxes of IκB to and from c0, respectively. As shown in Figure 4C, *flux_IκB_co:c1_* was periodically negative at D_protein_ of 10^−11^ m^2^/s, indicating net inward flux from c1 to c0. However, it was never negative at D_protein_ of 10^−13^ m^2^/s, reaching zero at equilibrium. This indicated that there was no net inward flow from c1 to c0 under these conditions. All these simulation results clearly demonstrated the existence of the backward flux to the nucleus, strongly supporting the “reservoir” hypothesis.

At D_protein_ of 10^−13^ m^2^/s, the total IκB, which was the integrated amount of IκB and its complex within the entire 1D volume (*∫(IκB + IKK•IκB•NFκB + IκB•NFκB + IκB_n_ + IκB•NFκB_n_)dν*), was lower than the initial level, while it was higher at D_protein_ of 10^−11^ m^2^/s (Figure S5). This demonstrated the degradation-dominant processes in IκB at D_protein_ of 10^−13^ m^2^/s and, because of this, the export of NF-κB_n_ and hence the “reset” were insufficient, reaching equilibrium and halting the oscillation.

### Oscillation of NF-κB_n.tot_ was rescued by the increase in D_IκB_ while keeping D for other proteins small

**Figure 5:**
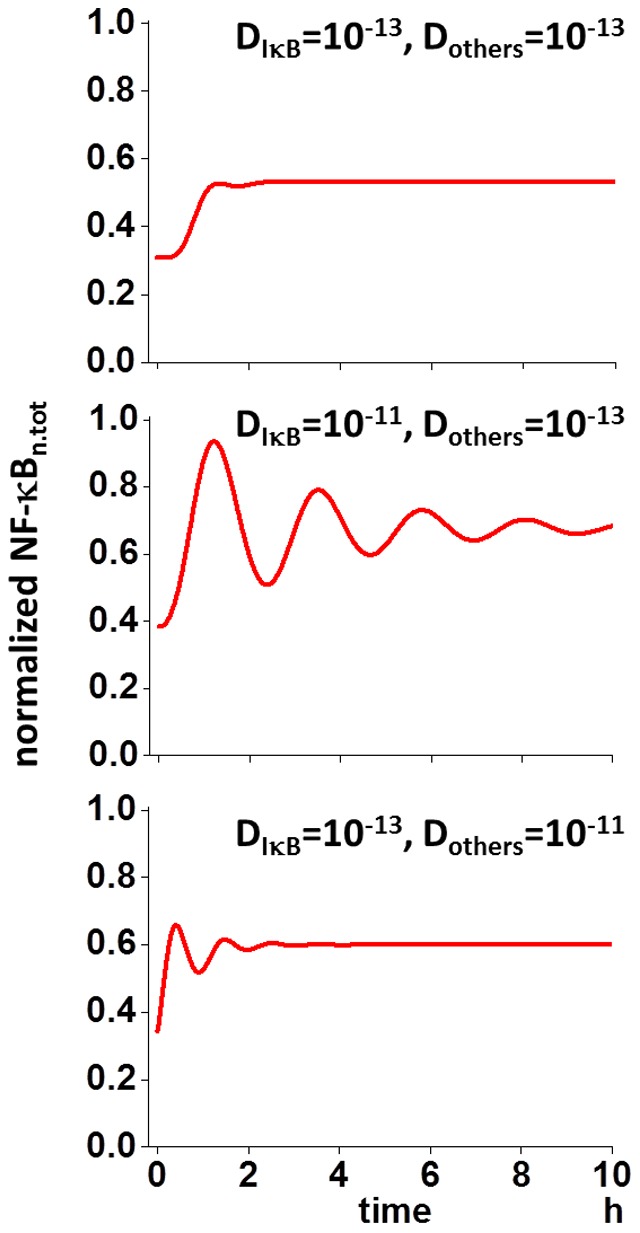
Rescue simulation for the oscillation of nuclear NF-κB. Oscillation was rescued by increasing D for IκB (10^−11^ m^2^/s) keeping D for other proteins small (10^−13^ m^2^/s, middle) from the non-oscillating condition where D for IκB and other proteins were 10^−13^ m^2^/s (top). In contrast, oscillation was not rescued when D for other proteins were increased while D for IκB was kept small (bottom) confirming the hypothesis shown in Figure.4A.

According to the “reservoir” hypothesis, in cases of increased diffusion coefficient for IκB (D_IκB_) while keeping D for other proteins (D_others_) small, the oscillation of NF-κB_n.tot_ should be rescued. In fact, this was the case if D_IκB_ was increased to 10^−11^ m^2^/s keeping D_others_ 10^−13^ m^2^/s (middle panel of Figure 5). If D_IκB_ was 10^−13^ while D_others_ was increased to 10^−11^ m^2^/s, the oscillation was not rescued (bottom panel). These results together with those shown in Figure 4 strongly support the view that the “reset” of NF-κB_n_ by IκB is the mechanism for the oscillation, and the restoration of nuclear IκB from the “reservoir” in the cytoplasm by a large IκB flux is crucial to the “reset” process.

### Simulated crowding of organelles around the nucleus alters the oscillation of NF-κB_n.tot_

The next question was how a change in the structure of cellular organelles affected the oscillation pattern of NF-κB_n.tot_. It was reported that mitochondria gather around the nucleus under conditions of hypoxia or a viral infection [37], [38]; the activation of NF-κB in response to hypoxia and viral infection has also been reported [5]–[10]. Although the diffusion coefficient is thought to be inherent to a protein, its effective value (D_eff_) can be changed by organelle crowding, and such a structural change will be biologically important for regulating intracellular signaling. In fact, Luby-Phelps et al. reported the reduction in the diffusion coefficient for molecules of larger size [46], [47]. From this result, they suggested the existence of structural obstacles to diffusion in cells. Dix et al. and Lin et al. discussed an effect of organelles as diffusion obstacles and a role in the signal transduction [48], [49]. They suggested that organelle crowding, subcellular structures (e.g. the budding neck of yeast), and sub-organelle structures (e.g. nuclear pores) acted as diffusion barriers controlling the spatio-temporal signaling. Dieteren et al. measured the diffusion coefficient in the mitochondria [50]. They concluded that intra-organelle structure, cristae in this case, hindered the diffusion. Furthermore, Mazel et al. reported on the effect of organelles on diffusion [51]. They reconstructed intracellular structures in a computer from images taken by electron microscopy, and ran computer simulations. They concluded that intracellular geometry limited diffusion. All these reports led us to hypothesize that organelle crowding in response to NF-κB-activating stimuli changed the oscillation pattern of NF-κB_n.tot_ by reducing D_eff_. We tested this possibility by running a set of simulations by changing the spatial distribution of organelles.

**Figure 6:**
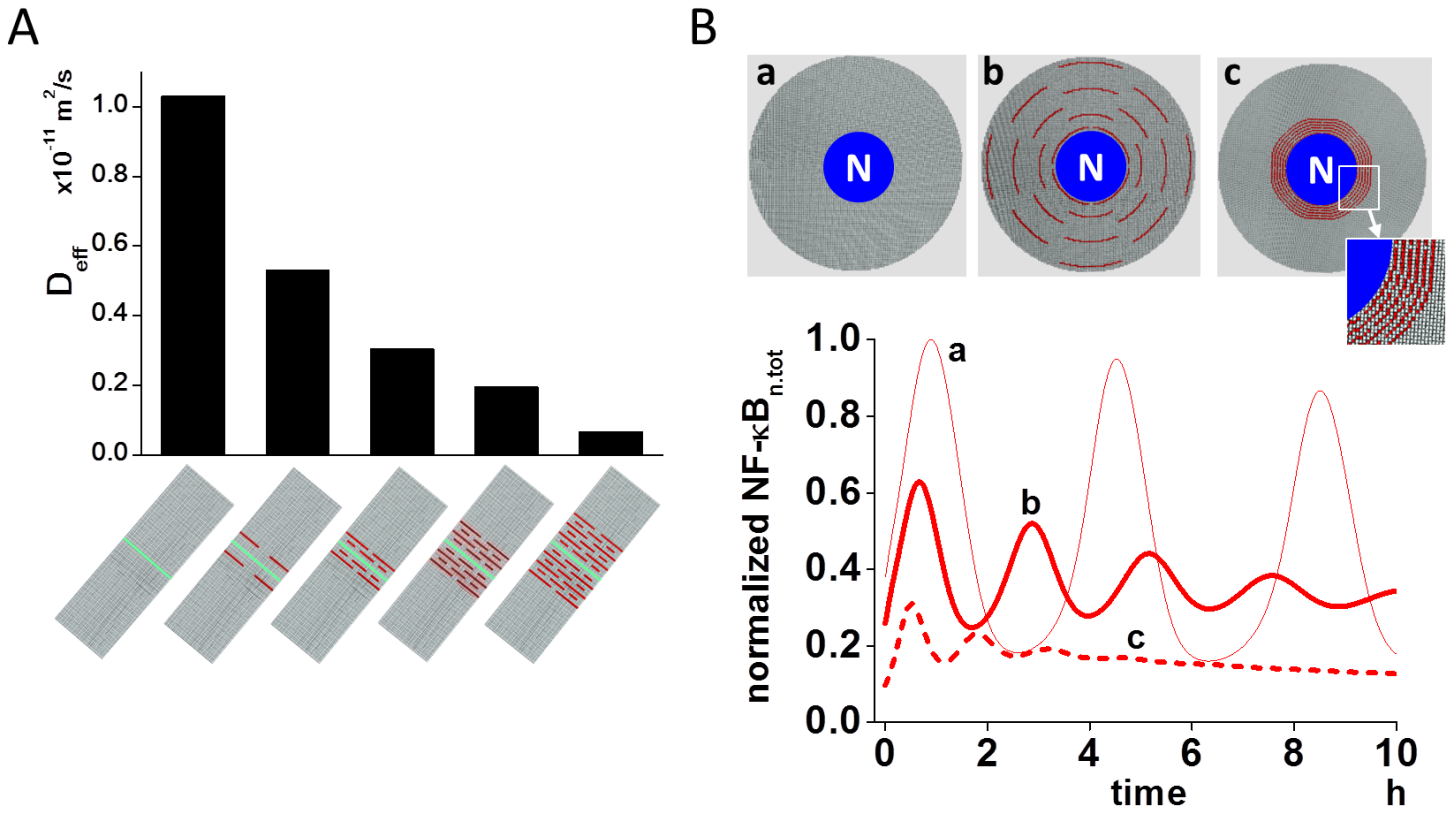
Organelle crowding around the nucleus alters the oscillation pattern of nuclear NF-κB. (A) Effective value of diffusion coefficient *D_eff_* was changed considerably by the addition of diffusion obstacles (short and long red lines). All diffusing substances were concentrated at the center (green line) at *t = 0*. D for all simulations was 10^−11^ m^2^/s, irrespectively of the presence or absence of diffusion obstacles. However, *D_eff_* was reduced by more than one order of magnitude by the increase in the population of diffusion obstacles. (B) The change in the oscillation pattern by the organelle crowding around the nucleus. Oscillation patterns were compared with three different densities of organelles. Oscillation pattern of NF-κB_n.tot_ without organelles is shown by thin red line (a). If organelles were added, the oscillation was heavily dampened (b, thick red line). If the organelles were crowded around nucleus, virtually no oscillation was observed (c, broken red line). Concentrations were normalized to the maximum value without organelles (case a).

First we tested how D_eff_ was altered by the change in organelle crowding. We increased the number of diffusion obstacles simulating the organelle crowding. To measure D_eff_ in the simulation we used Eq.7 (Cf. **Materials and Methods**). By using Eq.7, we could measure D_eff_ from the concentration of molecules at the origin and at a position x from the origin (Figure S6), and the estimated D using Eq.7 was in very good agreement with that used in the simulation (Cf. **Materials and Methods**). Simulation results showed that D_eff_ was reduced to less than 10% by the organelle crowding (Figure 6A). Green and red lines in Figure 6A indicate origin, where all diffusing species are concentrated at t = 0, and obstacles for diffusion, respectively.

To investigate further the effect of organelle crowding on NF-κB_n.tot_ oscillation, we constructed a 2D circular model cell with different crowding conditions of organelles (Figure 6B). The density (crowdedness) but neither the population nor the size of organelles was changed (Cf. Figure 6B, b and c). When organelles were added to the 2D model, the oscillation was heavily dampened in comparison to the situation where no organelles were added (thick and thin red continuous lines in Figure 6B). If organelles were crowded around the nucleus, virtually no oscillation was observed (red broken line in Figure 6B). Thus, our simulations show a possible change in the oscillation pattern of NF-κB_n.tot_ in response to the change in the organelle distribution. The same dampened oscillation by organelle crowding was also observed in the original 3D model (Figure S7).

## Discussion

We have studied a regulatory mechanism for the oscillation pattern of nuclear NF-κB by a diffusion coefficient. In the course of the analysis, we hypothesized that the “reset” of free NF-κB_n_ to the initial level by IκB was crucial for the oscillation. Insufficient “reset” results in the accumulation of IκB:NF-κB_n_ and NF-κB_n_ in the nucleus, and hence in the reduced amount of activated NF-κB in the cytoplasm. Since activated cytoplasmic NF-κB drives the oscillation by its transient translocation to the nucleus, the reduction of cytoplasmic NF-κB leads to the equilibrium halting the oscillation. Thus, the “reset” is inherently important for the oscillation of nuclear NF-κB. A large D contributed to the “reset” of free NF-κB_n_ by storing sufficient IκB within a “reservoir” in the cytoplasm that could subsequently be used to replenish the nucleus. The diffusion coefficient is thought to be inherent to the diffusing species. However, its effective value can be changed by the redistribution of organelles, because they act as obstacles to diffusion [51]. In fact our simulations showed the reduction in the effective diffusion coefficient and an alteration in the oscillation pattern of NF-κB_n.tot_ by the crowding of organelles around nucleus.

There has been discussion on whether the oscillation pattern of NF-κB regulates the gene expression profiles [52], [53]. Further experiments are still required before reaching any conclusion, and the present work was not intended to present results that could add to this debate. Rather, we wanted to show the possible phenomena and their mechanisms on the regulation of oscillation pattern of NF-κB by the diffusion coefficient, because NF-κB-activating stimuli are reported to change the distribution, population and density of organelles [37], [38]; the change in these spatial parameters can change the effective diffusion coefficient.

We have shown a possible change in the oscillation pattern of NF-κB_n.tot_ by the crowding of organelles. We ran simulations assuming mitochondria as to be the obstacles for diffusion, because they are reported to crowd around the nucleus under conditions of hypoxia or the presence of a viral infection [37], [38]. ER is also reported to be a quite dynamic organelle that frequently changes its structure [54], [55]. Thus, spatial redistribution and/or the change in the shape of ER would also affect the oscillation pattern of NF-κB together with mitochondria. To the best of our knowledge, there are no previous reports showing a possible relationship between NF-κB oscillation pattern and organelle crowding. Further experiments and simulations on the regulation of NF-κB oscillation pattern by organelle crowding will establish its role in gene expression profiles. In particular, 3D simulations with true intracellular space (TiCS), which is a computerized intracellular space extracted from imaging data of electron-microscopic resolution, is important in this respect, because TiCS provides an adequate level of information for the simulation of organelle redistribution [56].

We have focused on the role of the diffusion coefficient in the regulation of NF-κB oscillation pattern. We previously reported that nuclear transport, N/C ratio, and locus of protein synthesis are also involved in the control of the oscillation pattern of NF-κB [39]. Therefore, it is also important to reveal the mechanisms controlling the oscillation pattern by these spatial parameters and their biological significance.

In this study, we found that the “reset” of free NF-κB_n_ and the cytoplasmic “reservoir” for IκB are crucial for the oscillation of NF-κB_n.tot_. We did not see continued oscillation within the range of our simulation, because cumulative *Δflux* became positive at a later time after the start of the oscillation. However, there is a possibility of acquiring continuous oscillation by changing spatial and/or kinetic parameters. This might have a relationship with the constitutive activity of NF-κB in cancer.

## Materials and Methods

### Computational model

We constructed three spatio-temporal computational models of NF-κB oscillation. These included 3D, 2D, and 1D models. The 3D model was basically the same as that used in a previous report [39], and formed the basis of our study. The 2D model was used for the investigation of the effect of crowding of organelles. Chemical reactions used for the 3D and 2D models were the same as used in a previous report [39]. Briefly, the models for NF-κB activation comprised the formation of IKK:IκBα:NF-κB complex, the degradation of IκBα and subsequent nuclear transportation of NF-κB, NF-κB transcription of IκBα mRNA, IκBα protein synthesis, and the nuclear export of IκBα:NF-κB complex (Figure S1A). Chemical reactions in the 1D model were simplified to investigate the essence of the effect on the diffusion coefficient (Figure S1B). None of these models included all the molecular mechanisms shown in the **Introduction**, and aimed at extracting the phenomena and the mechanisms for the control of the NF-κB oscillation pattern by the diffusion coefficient.

The 3D spherical cell model with a diameter of 50 µm was divided into small cubic compartments (total 62,417) of identical size enabling reaction-diffusion simulations (Figure 1A). We used Fick's equation for simulating diffusion, which was combined with differential equations for chemical reactions. The central 8.3% compartments were assigned as the nucleus. In the 2D model, the diameter and the thickness of the model cell was 30 µm and 0.2 µm, respectively, which was divided into 18,033 cubic compartments with an edge length of 0.2 µm per cube. Organelles, which acted as diffusion obstacles, were constructed around the nucleus to investigate the effect of organelle crowding on the oscillation pattern of NF-κB (Figure 6B). In the 1D model, which was used for the analysis of the effect of the diffusion coefficient, there were 10 cubic compartments with an edge length of 5 µm per cube, and the rightmost red compartment c0 was assigned as the nucleus and nuclear membrane compartment (Figure 2A). Reaction schemes shown in Figure S1 were embedded in the corresponding region of the cytoplasm, nuclear membrane, and nucleus of the 3D, 2D and 1D models.

We employed the 1D model for efficient analyses, because there were only 1/6241.7^th^ compartments in 1D model compared to that in 3D model. For the simulation of organelle crowding, we used the 2D model with a much higher number of divisions into compartments, because we wanted to construct organelles with a finer spatial resolution than in the original 3D model.

All three models were constructed using A-Cell software [57], [58]. Models and all parameters used in the present study can be downloaded from http://www.ims.u-tokyo.ac.jp/mathcancer/A-Cell/index.html. Kinetic parameters used in our simulation are listed in Table S1 for the 3D, 2D, and 1D models.

### Simulations

Simulation programs in c language were automatically generated by A-Cell. We used the parallelized version by openMP for a multi-core CPU. Simulations were run on a Linux computer with Intel compiler. Initial conditions shown in Table S1 were for D_protein_ of 10^-11^ m^2^/s. Every time we changed D_protein_, we first acquired an equilibrium forcing IKK = 0, which ensured a resting state. Thereafter a simulation of NF-κB oscillation was run by setting concentrations acquired by the equilibration. Simulated concentrations of nuclear NF-κB were plotted in normalized values to the maximum at D_protein_ of 10^-11^ m^2^/s unless otherwise noted.

### Bifurcation Analysis

Traditionally, a bifurcation diagram is drawn at a quasi-equilibrium state, and is used extensively to show the change in the system behavior by a characteristic parameter. In the present analysis, we defined that NF-κB was oscillating if there was at least one peak and trough. Based on this definition, NF-κB was not in an equilibrium state. Therefore, our analysis was not the traditional bifurcation analysis. The reason why we did not follow traditional analysis was that the IκBα gene expression, which is important for the regulation of the oscillation pattern (Cf. main text), was expressed even with a single pulsatile stimulation [13]. If we performed the bifurcation analysis 20,000 sec after the start of the oscillation, we achieved almost the same diagram as shown in Figure 2B (Cf. Figure S2).

### Estimation of effective diffusion coefficient D_eff_

We began with the well-known diffusion equation shown below:



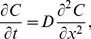
(5)


where *C* and *D* are concentration and diffusion coefficient, respectively. The analytical solution of Eq.5 in 1D space is



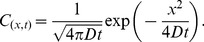
(6)


From Eq.6, we developed an equation for estimating diffusion coefficient as follows:



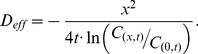
(7)


If we know the concentrations at the origin and at position *x* at time *t*, we can calculate *D_eff_*. We estimated *D_eff_* of 1.03×10^-11^ m^2^/s by measuring *C_(x,t)_* and *C_(0,t)_* in the simulation with known *x* and *t*, which was very close to that used in the simulation (10^-11^ m^2^/s). Thus we can estimate *D_eff_* reliably by using Eq.7 (Figures 6 and S6).

## Supporting Information

Figure S1 **Reaction scheme for 3D, 2D, and 1D simulation.** Reaction schemes for 3D and 2D simulations are the same as those in a previous report (A). The reaction schemes for 1D are simplified and aimed at revealing essential mechanisms for the regulation of oscillation pattern by the diffusion coefficient. For this purpose, a spontaneous decay of IKK was not involved. (B).
(TIF)

Figure S2 **Bifurcation diagrams: comparison of the first peak and trough and the first peak and trough 20,000 sec after the start of the oscillation.** The definition of the first peak and trough, and the first peak and trough after 20,000 sec are shown (A). Bifurcation diagram for the first peak and trough 20,000 sec after the start of the oscillation are shown in thick lines. It can clearly be seen that NF-κB_n.tot_ oscillates at D of higher that 10^−11^ m^2^/s. The diagram for the first peak and trough is shown in thin dashed lines (B).
(TIF)

Figure S3 **Bifurcation diagram in 3D model.** Bifurcation was also observed in the original 3D model, which was drawn for the first peak and trough 20,000 sec after the start of the oscillation.
(TIF)

Figure S4 **Cumulative** ***Δflux*** **analysis at D of 10^−13^ and 10^−9^ m^2^/s.** Magnified view of the time course of IκB at D of 10^−13^ m^2^/s shows a peak at the time indicated by an arrow (A). When D was 10^−9^ m^2^/s, the concentration of free NF-κB_n_ at the first trough was smaller than the initial level (gray arrow in the top panel of B). In parallel to this, the cumulative *Δflux* was negative at the first trough indicating a lower concentration than the initial level (gray arrow in the bottom panel of B). Red and blue broken lines indicate initial levels of free NF-κB_n_ and IκB_n_, respectively.
(TIF)

Figure S5 **Degradation-dominant process in small D condition.** Total IκB complex, which was the integrated amount of IκB and its complex within the entire 1D volume (*∫(IκB + IKK•IκB•NFκB + IκB•NFκB + IκB_n_ + IκB•NFκB_n_)dν*), was lower at equilibrium than the initial level (broken lines) at D_protein_ of 10^−13^ m^2^/s, while it was higher at D_protein_ of 10^−11^ m^2^/s. This indicated that at low D_protein_ condition the degradation dominated the de novo synthesis of IκB.
(TIF)

Figure S6 **Simulation for estimating effective diffusion coefficient.** To estimate the effective diffusion coefficient, *D_eff_*, a 2D rectangle space measuring 101 by 31 µm was divided into 101 and 31 small compartments allowing simulations of diffusion (top panel). All substances were concentrated in the central 31 compartments before the start of the simulation (green line in the top panel). The diffusion in this arrangement is essentially 1D. At *t* after the start of the simulation, substances were distributed as shown in the middle panel with higher (red) and lower (blue) concentration. The spatial profile at *t* is shown in the bottom panel, from which we can measure the concentrations at the center (*B_(0,t)_*) and at location *x* (*B_(x,t)_*), and we can estimate *D_eff_* using Eq.7 shown in the main text. We measured *D_eff_* with various population of obstacles (Cf. Figure 6A).
(TIF)

Figure S7 **Heavily dampened oscillation by the organelle crowding in 3D model.** The increased dampened oscillation caused by the organelle crowding was also observed in the original 3D model.
(TIF)

Table S1 **Parameter values for 3D, 2D and 1D simulations.** Kinetic parameter values are listed. Concentrations were for D_protein_ of 10^−11^ m^2^/s. Kinetic parameters are not the same for the 3D, 2D and 1D simulations, because, if we used the same parameters, the oscillation pattern of NF-κB_n.tot_ was highly different from that observed in the previous experiments [39]. Therefore, we determined different set of parameter values in order to acquire the identical oscillation pattern for 3D, 2D and 1D under control conditions. IKK for 3D, 2D and 1D simulations were embedded into a single compartment for ease of simulation. The IKK concentration in the list was averaged for all cytoplasmic compartments. Thus, the average concentration of IKK was the same for all 3D, 2D and 1D simulations. Since the diffusion of proteins is rapid and homogeneously distributed within a negligible time period after the start of the simulation [39], this initial setting of IKK in 3D, 2D, and 1D simulation has virtually no effect on the oscillation pattern of NF-κB. Prefix ‘n_’ indicates species in the nucleus.
(DOCX)
