# Supplementary figures and images for: Regulation of Nuclear NF-κB Oscillation by a Diffusion Coefficient and Its Biological Implications
Source: PLoS One. 2014 Oct 10;9(10):e109895. doi: 10.1371/journal.pone.0109895 (PMC4193834; doi:10.1371/journal.pone.0109895)

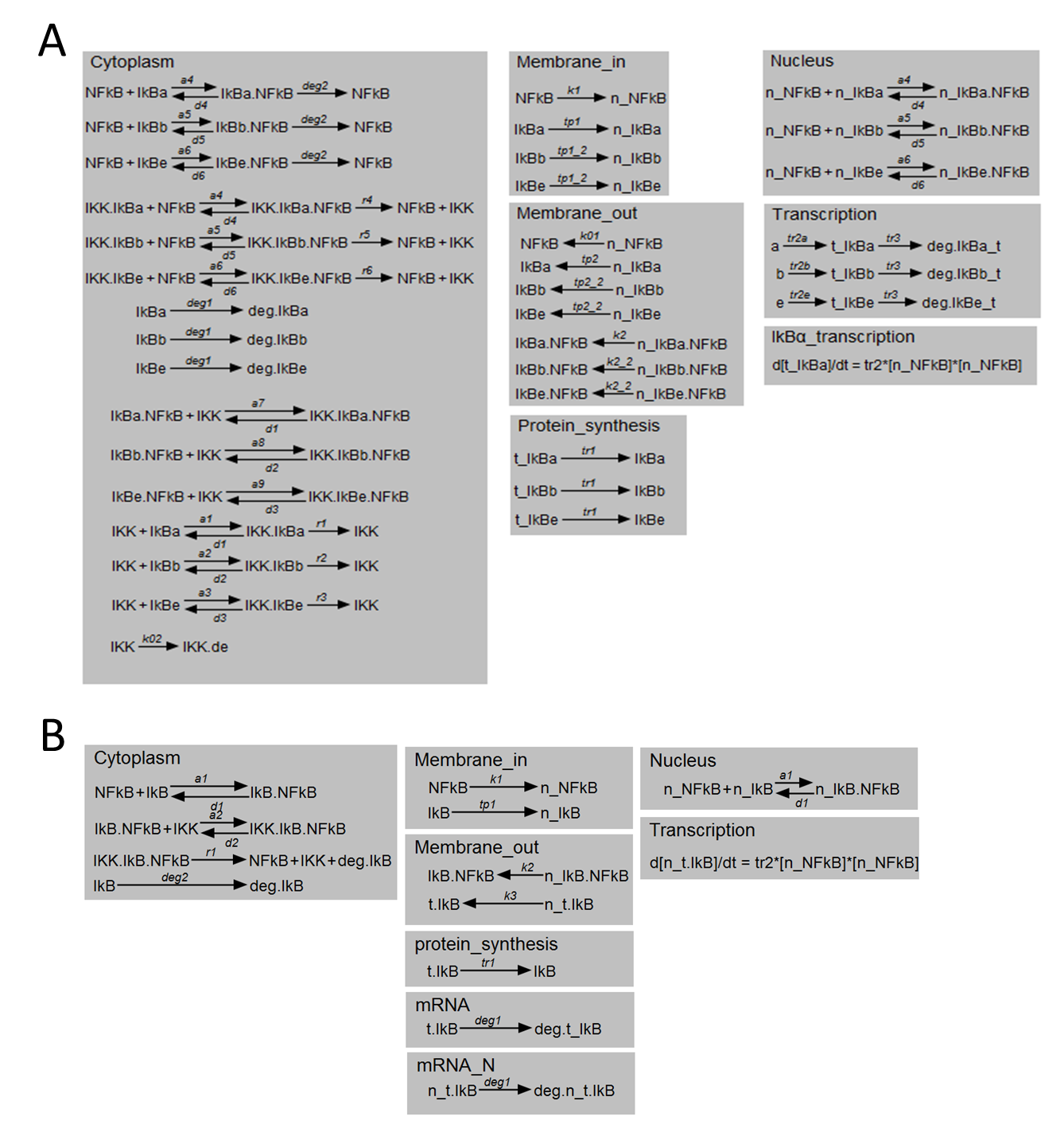

Supplement: Figure S1 — Reaction scheme for 3D, 2D, and 1D simulation. Reaction schemes for 3D and 2D simulations are the same as those in a previous report (A). The reaction schemes for 1D are simplified and aimed at revealing essential mechanisms for the regulation of oscillation pattern by the diffusion coefficient. For this purpose, a spontaneous decay of IKK was not involved. (B). (TIF) [file pone.0109895.s001.tif]

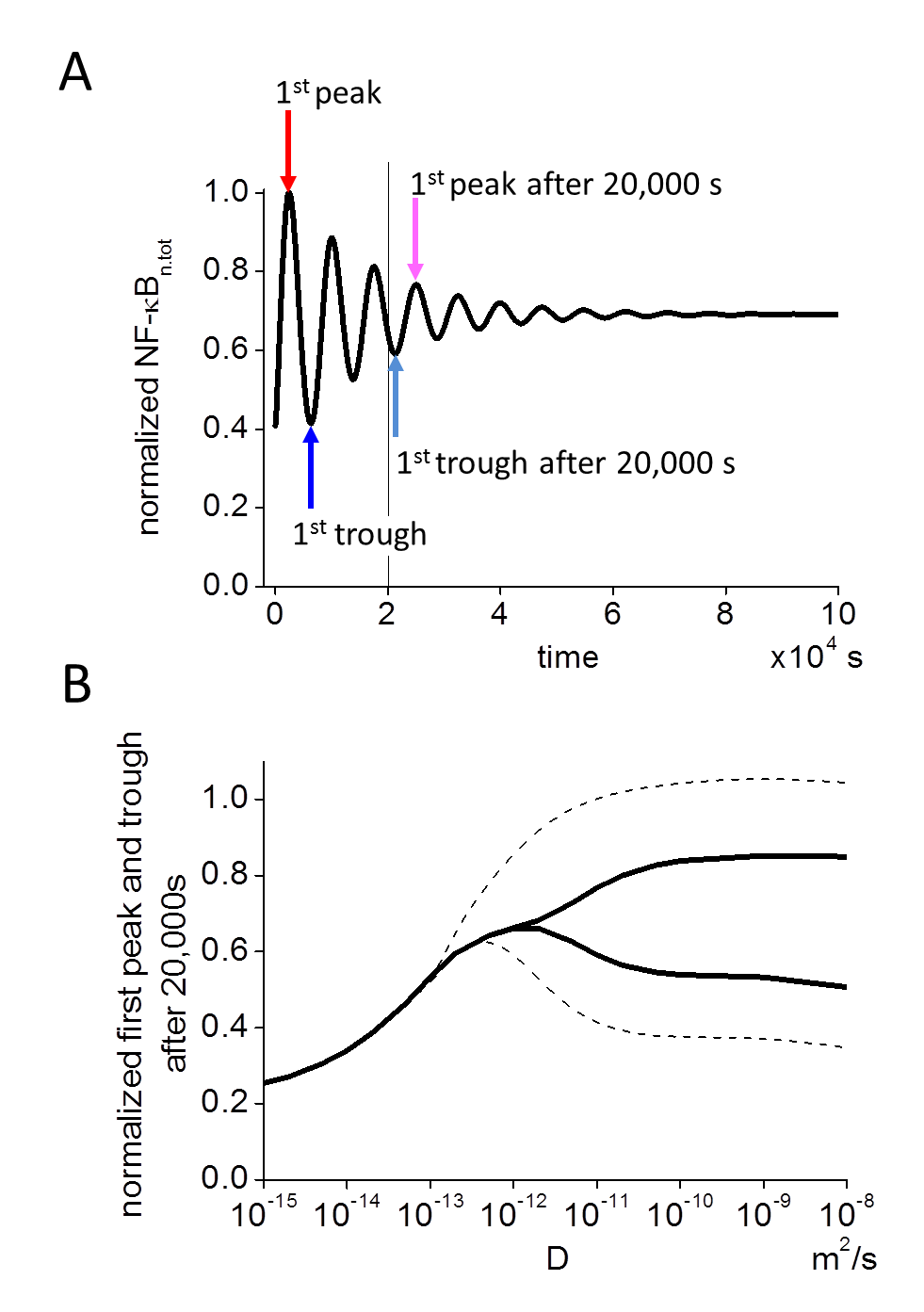

Supplement: Figure S2 — Bifurcation diagrams: comparison of the first peak and trough and the first peak and trough 20,000 sec after the start of the oscillation. The definition of the first peak and trough, and the first peak and trough after 20,000 sec are shown (A). Bifurcation diagram for the first peak and trough 20,000 sec after the start of the oscillation are shown in thick lines. It can clearly be seen that NF-κBn.tot oscillates at D of higher that 10−11 m2/s. The diagram for the first peak and trough is shown in thin dashed lines (B). (TIF) [file pone.0109895.s002.tif]

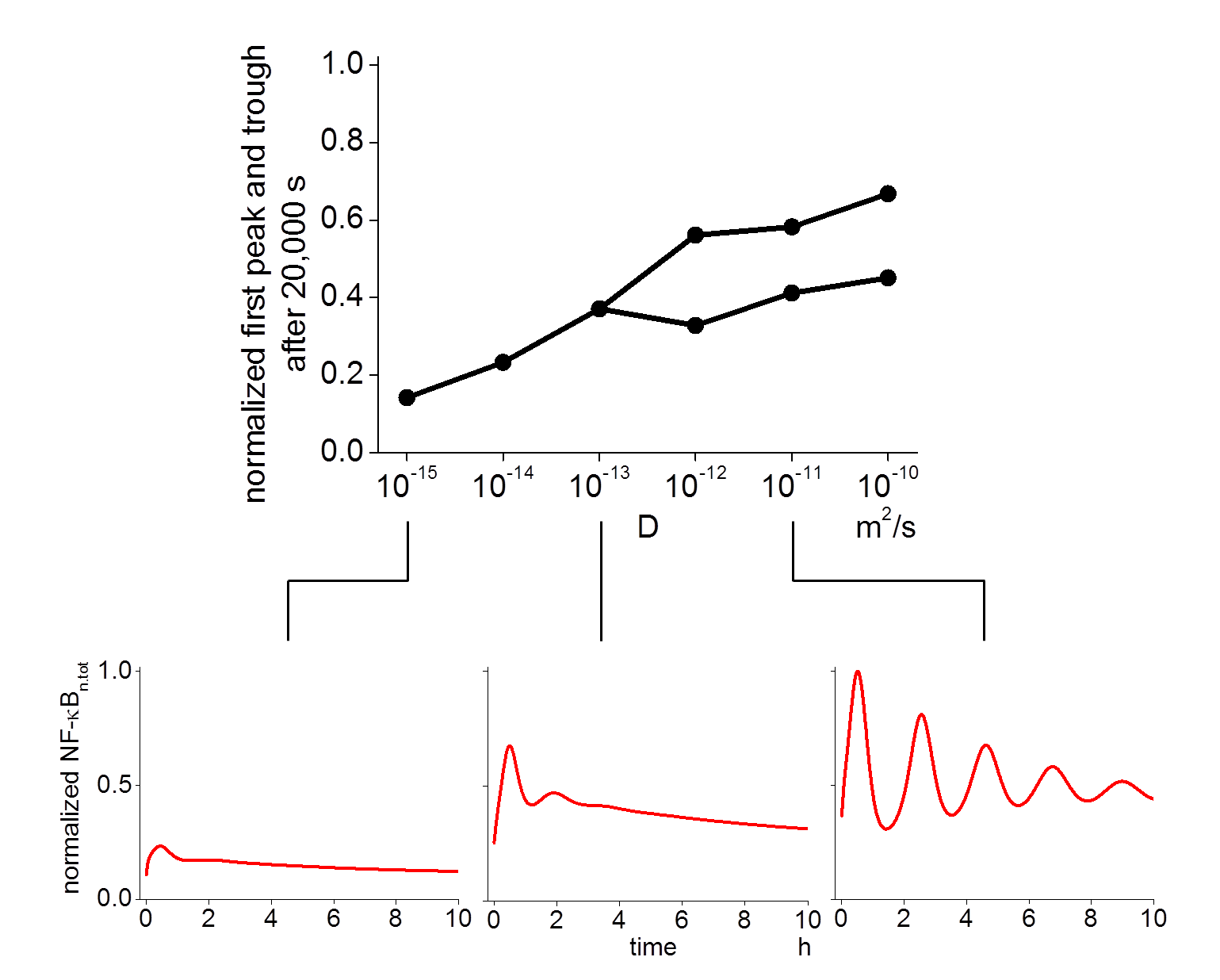

Supplement: Figure S3 — Bifurcation diagram in 3D model. Bifurcation was also observed in the original 3D model, which was drawn for the first peak and trough 20,000 sec after the start of the oscillation. (TIF) [file pone.0109895.s003.tif]

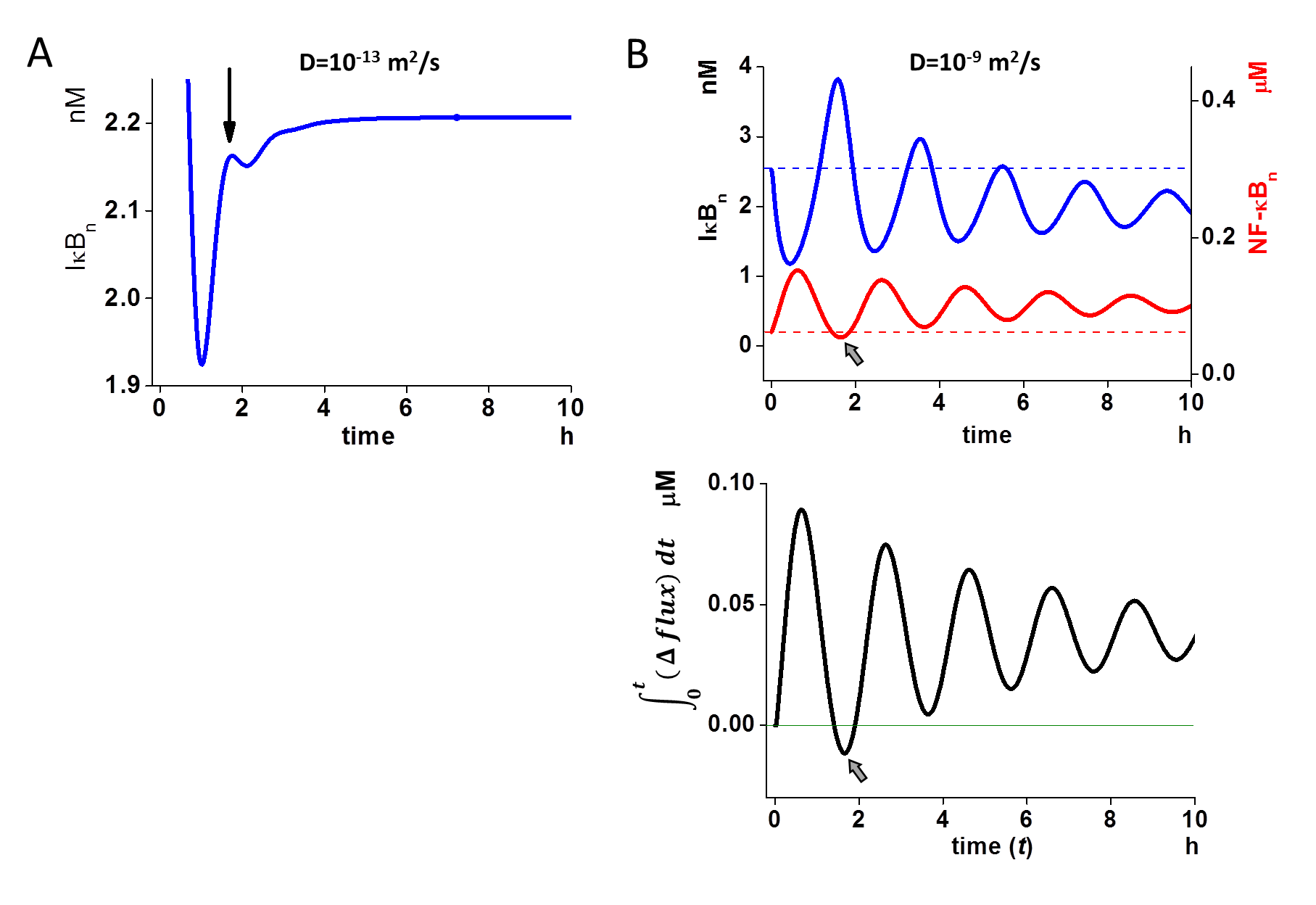

Supplement: Figure S4 — Cumulative Δflux analysis at D of 10−13 and 10−9 m2/s. Magnified view of the time course of IκB at D of 10−13 m2/s shows a peak at the time indicated by an arrow (A). When D was 10−9 m2/s, the concentration of free NF-κBn at the first trough was smaller than the initial level (gray arrow in the top panel of B). In parallel to this, the cumulative Δflux was negative at the first trough indicating a lower concentration than the initial level (gray arrow in the bottom panel of B). Red and blue broken lines indicate initial levels of free NF-κBn and IκBn, respectively. (TIF) [file pone.0109895.s004.tif]

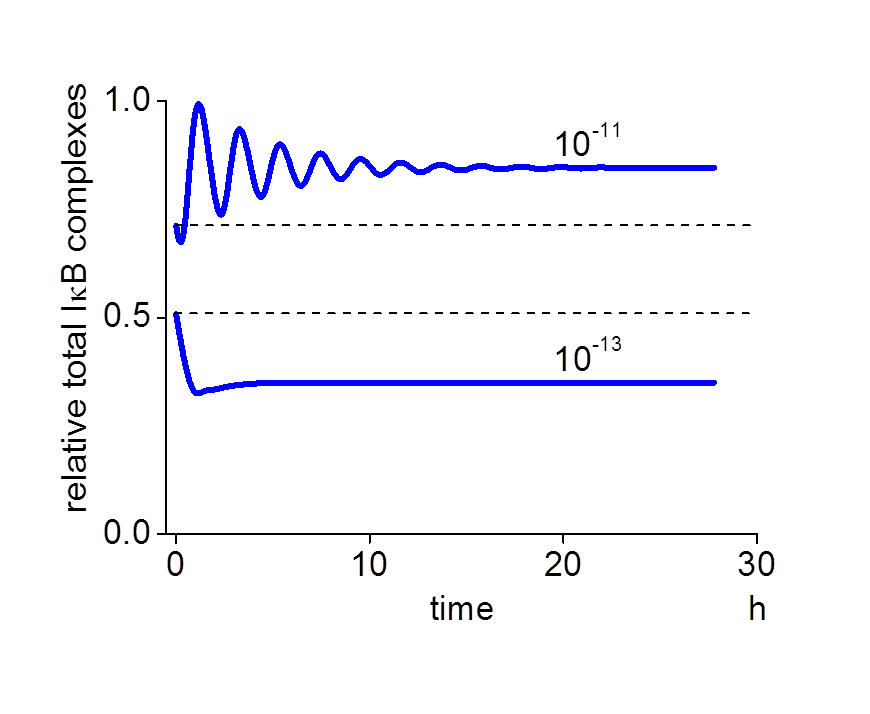

Supplement: Figure S5 — Degradation-dominant process in small D condition. Total IκB complex, which was the integrated amount of IκB and its complex within the entire 1D volume (∫(IκB + IKK•IκB•NFκB + IκB•NFκB + IκBn + IκB•NFκBn)dν), was lower at equilibrium than the initial level (broken lines) at Dprotein of 10−13 m2/s, while it was higher at Dprotein of 10−11 m2/s. This indicated that at low Dprotein condition the degradation dominated the de novo synthesis of IκB. (TIF) [file pone.0109895.s005.tif]

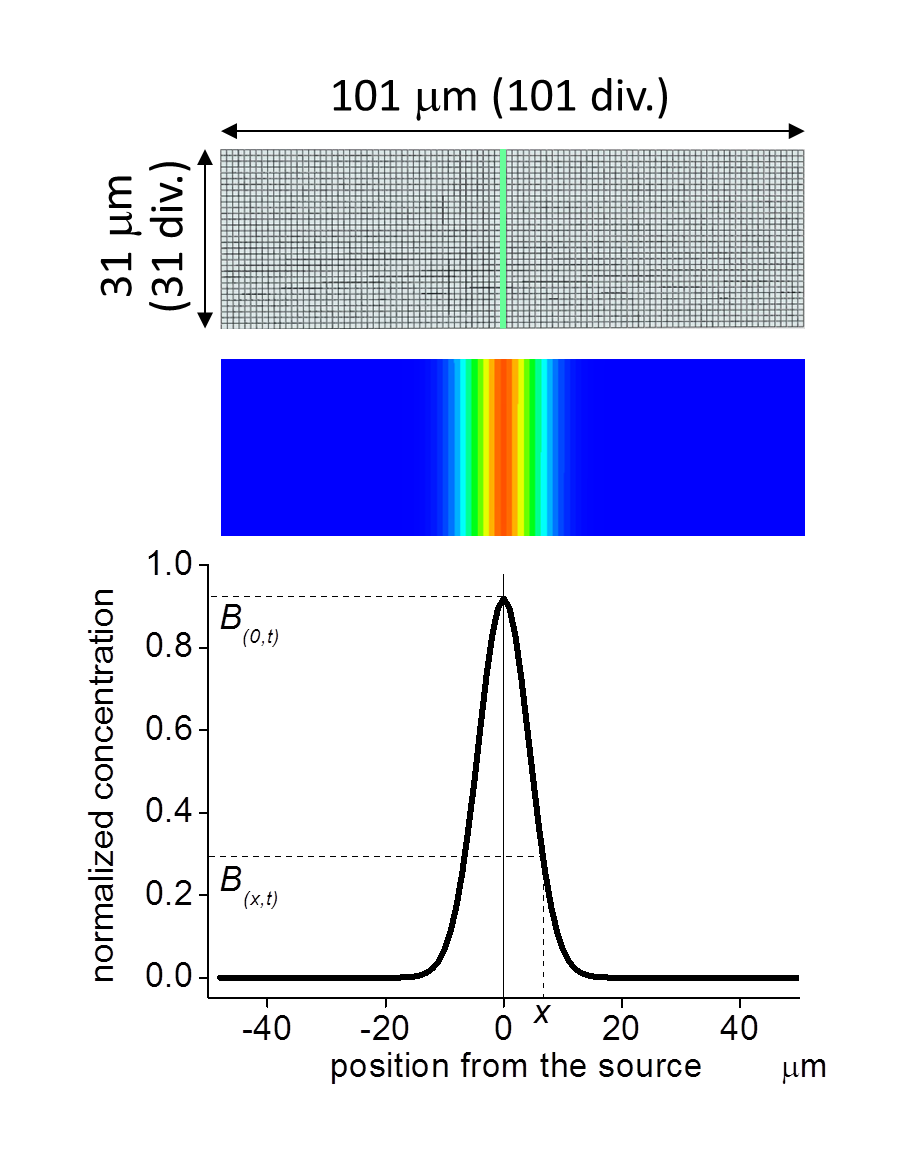

Supplement: Figure S6 — Simulation for estimating effective diffusion coefficient. To estimate the effective diffusion coefficient, Deff, a 2D rectangle space measuring 101 by 31 µm was divided into 101 and 31 small compartments allowing simulations of diffusion (top panel). All substances were concentrated in the central 31 compartments before the start of the simulation (green line in the top panel). The diffusion in this arrangement is essentially 1D. At t after the start of the simulation, substances were distributed as shown in the middle panel with higher (red) and lower (blue) concentration. The spatial profile at t is shown in the bottom panel, from which we can measure the concentrations at the center (B(0,t)) and at location x (B(x,t)), and we can estimate Deff using Eq.7 shown in the main text. We measured Deff with various population of obstacles (Cf. Figure 6A). (TIF) [file pone.0109895.s006.tif]

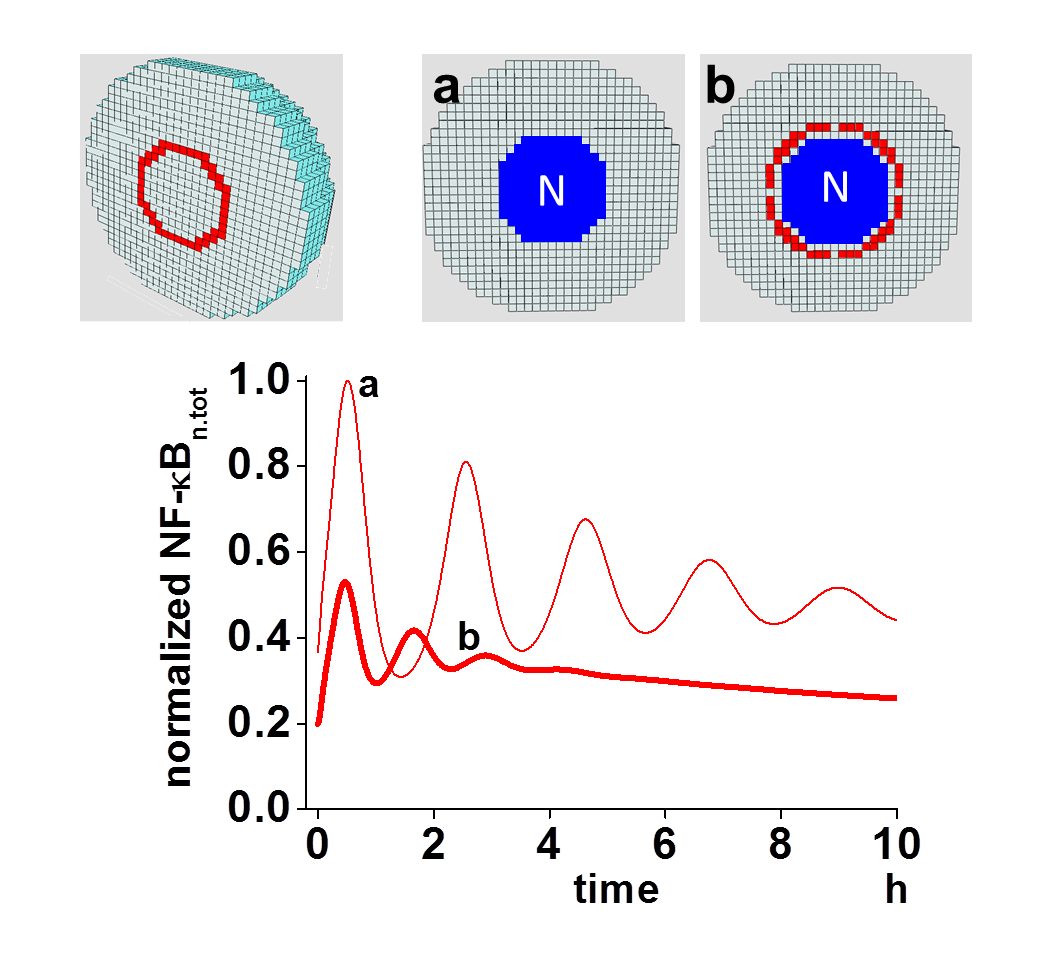

Supplement: Figure S7 — Heavily dampened oscillation by the organelle crowding in 3D model. The increased dampened oscillation caused by the organelle crowding was also observed in the original 3D model. (TIF) [file pone.0109895.s007.tif]
