# Supplementary material for: Regulation of Nuclear NF-κB Oscillation by a Diffusion Coefficient and Its Biological Implications
Source: PLoS One. 2014 Oct 10;9(10):e109895. doi: 10.1371/journal.pone.0109895 (PMC4193834; doi:10.1371/journal.pone.0109895)
Supplement: Table S1 — Parameter values for 3D, 2D and 1D simulations. Kinetic parameter values are listed. Concentrations were for Dprotein of 10−11 m2/s. Kinetic parameters are not the same for the 3D, 2D and 1D simulations, because, if we used the same parameters, the oscillation pattern of NF-κBn.tot was highly different from that observed in the previous experiments [39]. Therefore, we determined different set of parameter values in order to acquire the identical oscillation pattern for 3D, 2D and 1D under control conditions. IKK for 3D, 2D and 1D simulations were embedded into a single compartment for ease of simulation. The IKK concentration in the list was averaged for all cytoplasmic compartments. Thus, the average concentration of IKK was the same for all 3D, 2D and 1D simulations. Since the diffusion of proteins is rapid and homogeneously distributed within a negligible time period after the start of the simulation [39], this initial setting of IKK in 3D, 2D, and 1D simulation has virtually no effect on the oscillation pattern of NF-κB. Prefix ‘n_’ indicates species in the nucleus. (DOCX) [file pone.0109895.s008.docx]

**Table S1 Symbol values for 3D, 2D, and 1D simulations.**

| symbol | 3D | 2D | 1D | unit |  | symbol | 3D | 2D | 1D | unit |
| --- | --- | --- | --- | --- | --- | --- | --- | --- | --- | --- |
| a1 | 1.80x10^5^ | 1.80x10^5^ | 5.00x10^5^ | /M/s |  | tr1 | 4.00x10^-3^ | 4.00x10^-3^ | 4.00x10^-2^ | /s |
| a2 | 6.00x10^3^ | 6.00x10^3^ | 1.85x10^5^ | /M/s |  | tr2 | 1.70x10^6^ | 1.70x10^6^ | 1.70x10^5^ | /M/s |
| a3 | 9.00x10^3^ | 9.00x10^3^ | - | /M/s |  | a･tr2a | 1.50x10^-12^ | 1.50x10^-12^ | - | M/s |
| a4 | 1.00x10^5^ | 1.00x10^5^ | - | /M/s |  | b･tr2b | 2.30x10^-13^ | 2.30x10^-13^ | - | M/s |
| a5 | 5.00x10^5^ | 5.00x10^5^ | - | /M/s |  | e･tr2e | 1.70x10^-13^ | 1.70x10^-13^ | - | M/s |
| a6 | 5.00x10^5^ | 5.00x10^5^ | - | /M/s |  | tr3 | 2.80x10^-4^ | 2.80x10^-4^ | 2.80x10^-2^ | /s |
| a7 | 3.70x10^5^ | 3.70 x10^5^ | - | /M/s |  | IKK | 2.00x10^-9^ | 2.00x10^-9^ | 2.00x10^-9^ | M |
| a8 | 4.80x10^4^ | 4.80x10^4^ | - | /M/s |  | IKK.IkBa | 0 | 0 | 0 | M |
| a9 | 7.00x10^4^ | 7.00x10^4^ | - | /M/s |  | IKK.IkBa.NFkB | 0 | 0 | 0 | M |
| d1 | 1.25x10^-3^ | 1.25x10^-3^ | 5.00x10^-4^ | /s |  | IKK.IkBb | 0 | 0 | - | M |
| d2 | 1.75x10^-3^ | 1.75x10^-3^ | 1.25x10^-3^ | /s |  | IKK.IkBb.NFkB | 0 | 0 | - | M |
| d3 | 1.75x10^-3^ | 1.75x10^-3^ | - | /s |  | IKK.IkBe | 0 | 0 | - | M |
| d4 | 5.00x10^-4^ | 5.00x10^-4^ | - | /s |  | IKK.IkBe.NFkB | 0 | 0 | - | M |
| d5 | 5.00x10^-4^ | 5.00x10^-4^ | - | /s |  | IkBa | 2.87x10^-7^ | 2.42x10^-7^ | 5.68x10^-7^ | M |
| d6 | 5.00x10^-4^ | 5.00x10^-4^ | - | /s |  | IkBa.NFkB | 7.79x10^-8^ | 7.05x10^-8^ | 2.91x10^-7^ | M |
| deg1 | 1.00x10^-4^ | 1.00x10^-4^ | 2.80x10^-4^ | /s |  | IkBb | 7.23x10^-11^ | 8.91x10^-11^ | - | M |
| deg2 | 2.10x10^-5^ | 2.10x10^-5^ | 1.00x10^-4^ | /s |  | IkBb.NFkB | 8.31x10^-11^ | 1.12x10^-10^ | - | M |
| k01 | 1.60x10^-3^ | 4.80x10^-3^ | - | /s |  | IkBe | 5.34x10^-11^ | 6.58x10^-11^ | - | M |
| k02 | 8.30x10^-5^ | 8.30x10^-5^ | - | /s |  | IkBe.NFkB | 6.14x10^-11^ | 8.31x10^-11^ | - | M |
| k1 | 1.80x10^-1^ | 5.40x10^-1^ | 1.80x10^-1^ | /s |  | NFkB | 1.07x10^-9^ | 1.08x10^-9^ | 3.78x10^-10^ | M |
| k2 | 2.80x10^-2^ | 8.40x10^-2^ | 2.80x10^-2^ | /s |  | n_IkBa | 4.00x10^-8^ | 2.35x10^-8^ | 2.39x10^-9^ | M |
| k2_2 | 1.12x10^-1^ | 3.36x10^-1^ | - | /s |  | n_IkBa.NFkB | 5.59x10^-9^ | 4.87x10^-9^ | 2.44x10^-9^ | M |
| k3 | - | - | 2.80x10^-2^ | /s |  | n_IkBb | 1.07x10^-12^ | 8.79x10^-13^ | - | M |
| r1 | 3.70x10^-2^ | 3.70x10^-2^ | 3.70x10^-1^ | /s |  | n_IkBb.NFkB | 1.93x10^-13^ | 2.38x10^-13^ | - | M |
| r2 | 1.50x10^-2^ | 1.50x10^-2^ | - | /s |  | n_IkBe | 7.94x10^-13^ | 6.50x10^-13^ | - | M |
| r3 | 2.20x10^-2^ | 2.20x10^-2^ | - | /s |  | n_IkBe.NFkB | 1.43x10^-13^ | 1.76x10^-13^ | - | M |
| r4 | 1.85x10^-1^ | 1.85x10^-1^ | - | /s |  | n_NFkB | 2.14x10^-8^ | 1.78x10^-8^ | 5.81x10^-8^ | M |
| r5 | 7.50x10^-2^ | 7.50x10^-2^ | - | /s |  | t_IkBa | 1.71x10^-7^ | 3.44x10^-7^ | 1.41x10^-8^ | M |
| r6 | 1.10x10^-1^ | 1.10x10^-1^ | - | /s |  | t_IkBb | 5.05x10^-11^ | 1.47x10^-10^ | - | M |
| tp1 | 6.00x10^-4^ | 1.80x10^-3^ | 1.20x10^-4^ | /s |  | t_IkBe | 3.73x10^-11^ | 1.09x10^-10^ | - | M |
| tp1_2 | 3.00x10^-4^ | 9.00 x10^-4^ | - | /s |  | n_t_IkBa | 5.68x10^-8^ | 6.63x10^-8^ | 2.08 x10^-8^ | M |
| tp2 | 4.00x10^-4^ | 1.20x10^-3^ | - | /s |  | n_t_IkBb | 1.68x10^-11^ | 2.84x10^-11^ | - | M |
| tp2_2 | 2.00x10^-4^ | 6.00x10^-4^ | - | /s |  | n_t_IkBe | 1.24x10^-11^ | 2.10x10^-11^ | - | M |
| tp3 | 2.80 x10^-2^ | 8.40 x10^-2^ |  | /s |  |  |  |  |  |  |
